# Supplementary material for: Service user experiences and views regarding telemental health during the COVID-19 pandemic: A co-produced framework analysis
Source: PLoS One. 2021 Sep 16;16(9):e0257270. doi: 10.1371/journal.pone.0257270 (PMC8445423; doi:10.1371/journal.pone.0257270)
Supplement: Attachment — Submitted filename: Response to reviewers .pdf [file pone.0257270.s003.pdf]

Dear Prof. Myers,

We would like to thank you for sending us the comments made by the reviewers on our manuscript *Service user experiences and views regarding telemental health during the COVID-19 pandemic: a co-produced framework analysis* as these have strengthened the manuscript. We include a point-by-point response to their feedback below.

REVIEWER 1

- *Thank you for your article. It was a great pleasure to review it. The article addresses a crucial and actual topic. The participatory methodology utilized is exciting and enhances the research objectives. The results are thoroughly described, the discussion addresses the proposed questions and the results, and the implications suggested are meaningful. I recommend to publish the article as it stands.*

Thank you very much for your positive feedback.

REVIEWER 2

Thank you for your recommendations, we have addressed each one and outline below:

- *There are some minor grammatical errors in the abstract that should be attended to*

We thoroughly reviewed the abstract and have made some grammatical changes.

- *Please specify number of interviews in abstract*

We added this to the findings section of the abstract.

- *Background: greater attention to context of COVID-19 in the UK and how this impacted on care delivery is needed so that readers outside the UK can better understand the need for these service changes*

We added some information about key dates and changes experienced in the UK in the introduction page 3.

- *In the methods please report according to COREQ or similar standard reporting guidelines for qualitative research- this is a journal requirement*

We have completed a COREQ checklist to reflect compliance and attach it as supporting material.

- *How was consent for the interviews managed: remotely or in person*

Consent was obtained audio recorded remotely (specified now on page 5).

- *Table 1- some of the categories are hard to follow for an international reader- the urban/rural distinction is not clear for example. Would a town be urban or rural?*

Categories were self-defined. We have added a note to reflect this below Table 1.

- *Table 2- please add %*

Added now.

- *At what point in the pandemic did service users access the services and organisations described in Table 2 and 3. I may have missed it, but I think it may be useful to foreground more at what point in the pandemic service users were interviewed and the time period in the pandemic that they were asked to reflect on. Their experiences of mental health services may have been very different in a "hard" lockdown compared to a softer lockdown or between waves of infection. This links back to point 3.*

Services were accessed during periods of lockdown or social distance regulations in the UK in 2020, this is now mentioned in page 6 and under Table 3. We mention the potential differences in perceptions at different timepoints in in the pandemic in the limitations page 18.

- *The results are very long as there is room to consolidate these by removing some of the quotes, especially where there is repetition.*

We shortened or removed some of the quotes to make the results more concise.

- *Discussion. Any thoughts about whether these findings are relevant for settings outside of the UK?*

We have added some reflections around this in the discussion page 19.
